# Accommodation with anticancer drug shortage: A Lebanese harmful solution

**DOI:** 10.3389/fonc.2022.1055113

**Published:** 2023-01-18

**Authors:** Clarisse Kattan, Joseph Kattan

**Affiliations:** Department of Hematology-Oncology, Hotel-Dieu de France University Hospital, Faculty of Medicine, Saint Joseph University, Beirut, Lebanon

**Keywords:** drug shortage, standard of care, economic crisis, patient safety, rebound effect

## The current economic crisis in Lebanon

Since the end of 2019, an accumulation of multiple nested crises has been occurring in Lebanon, starting with the political revolution against the corrupt government, to the COVID-19 pandemic, to the explosion of the port of Beirut in August 2020, and to the economic crisis, resulting in a totally bankrupt country. (1)

The economic crisis is mostly the result of long-lasting political corruption that further resulted in local currency depreciation in 2020. The Lebanese pound devaluation led to the ruin of the central national bank resulting in the destabilization of the healthcare system. The detrimental impact of the economic crisis on the national healthcare system reached three levels: the shortage of imported drugs, collapse of third-party payment systems, and migration of healthcare professionals such as nurses and physicians seeking better job opportunities abroad (2).

## Lebanese healthcare system before the crisis

Lebanon has been consistently recognized as one of the most developed healthcare systems in the Middle East and North Africa through its world-renowned physicians and healthcare professionals. It has an excellent private hospital infrastructure and has always been a pioneer in applying innovative treatments and novel equipment. Additionally, almost total coverage of imported anticancer drugs by insurance companies and/or by the Ministry of Public Health offers all Lebanese cancer patients the latest generation of drugs and equity to access state-of-the-art healthcare (3).

## Failure of solution implementation by the government

In an attempt to contain the economic crisis, the Central Bank issued, at the end of 2019, an interim measure to subsidize the importation of anticancer medications. To do that, it provided 85% of the foreign currency needed for drug importation at the previously established official exchange rate (LBP 1,500.00 for USD 1.00 instead of the current value of LBP 40,000.00) (4). However, with the continuous currency deterioration, the smuggling of subsidized medications from Lebanon to other countries, and the stockpiling of medications by individuals and local warehouses, the subsidization strategy has led to frequent stock depletion and delays in drug importation. In parallel, illegal trafficking of counterfeit drugs has benefitted from corrupt dealers in black markets. Patients’ desperate need for expensive drugs such as immune checkpoint inhibitors and antiangiogenesis has been exploited by mafia groups. Most patients, being aware of this situation, have had no choice but to succumb to obtaining drugs illegally.

## Oncologists’ coping solutions

Currently, resilient oncologists who remain in the country are living a nightmare, facing a monthly reduction in their income and the impossibility of doing money transfers for renewing memberships or paying publishing fees secondary to the policy of capital control. However, to help overcome drug shortages and for the sake of their patients, oncologists are tempted by unorthodox approaches such as using on/off prescriptions, switching between different brands of the same drug class, using suboptimal drug dosages, and even deviating from the recommended mode of intake of some drugs.

### On/off prescription policy

The on/off prescription policy means that patients are only able to take the drug when it is available. This strategy mainly applies to expensive oral targeted drugs such as cyclin-dependent kinase (CDK) 4/6 inhibitors, new generation antiandrogens, and tyrosine kinase inhibitors (TKIs), and also intravenous drugs such as antiangiogenetic agents and immunotherapy drugs. It is not clear how harmful the on/off policy is. In our current practice, we have encountered a huge number of patients with metastatic breast cancer treated with a combination of CDK 4/6 inhibitors and either aromatase inhibitor or fulvestrant, who were unable to maintain a daily therapy longer than 2 consecutive months because of CDK 4/6 unavailability. As described by Hurvitz et al., when patients stopped taking a CDK 4/6 inhibitor, such as abemaciclib, for more than 4 days, Ki67 rebounded in 69% of the tumors compared with patients who remained on the drug (5). This rebound effect was also described in the literature with different TKIs (6). The use of cabozantinib, for instance, a multi-TKI given for locally advanced rearranged during transfection (RET)-mutated medullary carcinoma (7), achieved a remarkable response when given to one of our patients for 3 consecutive months. However, the disease progressed locally after the drug stopped being taken due to its unavailability and was deemed refractory when drug intake was resumed after 4 months. Similar cases were seen in patients who had been receiving the new generation of antiandrogens, such as enzalutamide given for metastatic castrate-resistant prostate cancer.

### Switching of brands in the same drug class

Switching brands based on accessibility is also being practiced, such as moving from one CDK 4/6 inhibitor to another in metastatic hormone-positive HER2 negative breast cancer, or by replacing enzalutamide with abiraterone acetate in metastatic castrate-resistant prostate cancer. These maneuvers, unlike biosimilars, are not recommended in the absence of any evidence of interchangeability between drugs with different mechanisms of action (8).

### Use of suboptimal drug doses

Underdosage is also being implemented by oncologists as an attempt to reduce drug expenses. Indeed, multiple expensive cytotoxic drugs such as nab-paclitaxel, cabazitaxel, vinflunine, pemetrexed, and azacitidine have been subject to unrecommended dose reduction. However, coping with the recommended dosage or even intensified dosage has been reported to be more effective with cytotoxic drugs (9).

### Deviation from the recommended mode of drug intake

Deviating from intake recommendations is also occasionally imposed. The oral anti-bcl2 venetoclax in relapsed chronic lymphocytic lymphoma (CLL) is recommended, starting with a ramp up schedule to avoid tumor lysis syndrome (10). Dosage is recommended to be increased weekly from 20 mg per day in the first week to reach 400 mg gradually. One of our CLL patients, a 60-year-old woman who was otherwise healthy, was being treated with multiple lines of chemotherapy and immunotherapy. Following this, salvage therapy with a combination of rituximab and venetoclax was prescribed according to the MURANO regimen (11). Venetoclax was not available, and so she received three cycles of rituximab alone, leading to a reduction in the volume of the tumor with the complete disappearance of palpable lymph nodes and normalization of the leucocyte count. However, the patient’s condition, her performance status, and her anemia did not improve. It was judged appropriate to introduce venetoclax, regardless of the cost. At our pharmacy, we found an abandoned 100-mg box of venetoclax that was still in date. We prescribed 100 mg of the drug to be taken every other day with a weekly escalation to reach 400 mg, with hydration and anti-uric acid precautions. However, the patient was admitted to the emergency room on the second day, after the first pill, with hyperkalemia, renal insufficiency, hyperuricemia, and pancreatitis. Fortunately, her symptoms were successfully managed during a 1-week stay at the intensive care unit after four hemodialysis sessions.

## Future solutions and hopes

It is well known that the high price of anticancer drugs limits treatment options for patients, harming them and society, especially in economically developing nations where such drugs are unaffordable (12). Imported generics, cheaper than branded drugs, could help lower drug bills (13). Importation of generic drugs from cheap manufacturers, such as from India, could be an essential part of the solution once quality control is implemented (14). In addition, manufacturing oncology drugs in local factories could help save money, but this would be difficult to realize in the short term. Donations from non-governmental organizations (NGOs) or the United Nations (UN) could also be helpful. However, the priorities of developed countries are more oriented toward supporting the Ukraine population rather than other developing countries. (15)

The Ministry of Public Health must urgently face this challenging situation by defining a new policy for drug importation, taking into account the type of drug and the country of origin, and must also fight corruption and prevent smuggling, stockpiling, and illegal drug trafficking.

## Conclusions

At present, Lebanese oncologists are extremely limited in their treatment options because expensive and essential oncology drugs are lacking. Standards of care are frequently not observed, putting cancer patients in critical and life-threatening situations. Attempts by oncologists to accommodate and circumvent drug shortages, based on good intentions and common sense but not on evidence, present more harm than solutions. However, until solutions are implemented by the Ministry of Public Health, this risky behavior remains unavoidable.

## Author contributions

JK: concept, objectives, and conclusions; CK: references and language improvement. All authors contributed to the article and approved the submitted version.

## References

[1] Mansour HA Bitar E Fares Y Makdessi A Maalouf A ElGhoul M. The Beirut port explosion: injury trends from a mass survey of emergency admissions. Lancet Lond Engl (2021) 398(10294):21–2. doi: 10.1016/S0140-6736(21)01246-0 34217389

[2] Lebanon: Scarce supplies of fuel and medicine push health system to the brink | doctors without borders . USA. Available at: https://www.doctorswithoutborders.org/latest/lebanon-scarce-supplies-fuel-and-medicine-push-health-system-brink (Accessed August 30, 2022).

[3] Elias F Khuri FR Adib SM Karam R Harb H Awar M. Financial burden of cancer drug treatment in Lebanon. Asian Pac J Cancer Prev APJCP (2016) 17(7):3173–7. 27509947

[4] Lebanon: Government recklessness in medication subsidy reform violates right to health and life . Amnesty International. Available at: https://www.amnesty.org/en/latest/news/2021/12/lebanon-government-recklessness-in-medication-subsidy-reform-violates-right-to-health-and-life/ (Accessed August 30, 2022).

[5] Hurvitz SA Martin M Press MF Chan D Fernandez-Abad M Petru E. Potent cell-cycle inhibition and upregulation of immune response with abemaciclib and anastrozole in neoMONARCH, phase II neoadjuvant study in HR+/HER2– breast cancer. Clin Cancer Res Off J Am Assoc Cancer Res (2020) 26(3):566–80. doi: 10.1158/1078-0432.CCR-19-1425 PMC7498177 31615937

[6] Pupo E Ducano N Lupo B Vigna E Avanzato D Perera T. Rebound effects caused by withdrawal of MET kinase inhibitor are quenched by a MET therapeutic antibody. Cancer Res (2016) 76(17):5019–29. doi: 10.1158/0008-5472.CAN-15-3107 27364553

[7] Colombo JR Wein RO. Cabozantinib for progressive metastatic medullary thyroid cancer: a review. Ther Clin Risk Manag (2014) 10:395–404. doi: 10.2147/TCRM.S46041 24920914 PMC4043815

[8] Nahleh Z Lyman GH Schilsky RL Peterson D Tagawa S Chavez- MacGregor. Use of biosimilar medications in oncology. JCO Oncol Pract (2022) 18(3):177–86. doi: 10.1200/OP.21.00771 35041524

[9] Nielson CM Bylsma LC Fryzek JP Saad HA Crawford J. Relative dose intensity of chemotherapy and survival in patients with advanced stage solid tumor cancer: A systematic review and meta-analysis. Oncologist (2021) 26(9):e1609–18. doi: 10.1002/onco.13822 PMC8417866 33973301

[10] Seymour J. Assessment of tumor lysis syndrome in patients with chronic lymphocytic leukemia treated with venetoclax in the clinical trial and post-marketing settings, in: ASH (2020). Available at: https://ash.confex.com/ash/2020/webprogram/Paper134938.html (Accessed August 30, 2022).

[11] Seymour JF Kipps TJ Eichhorst B Hillman P D'Rozario J Assouline S. Venetoclax–rituximab in relapsed or refractory chronic lymphocytic leukemia. N Engl J Med (2018) 378(12):1107–20. doi: 10.1056/NEJMoa1713976 29562156

[12] Prasad V De Jesús K Mailankody S. The high price of anticancer drugs: origins, implications, barriers, solutions. Nat Rev Clin Oncol (2017) 14(6):381–90. doi: 10.1038/nrclinonc.2017.31 28290490

[13] Cheung WY Kornelsen EA Mittmann N Leighi NB Cheung M Chan KK. The economic impact of the transition from branded to generic oncology drugs. Curr Oncol (2019) 26(2):89–93. doi: 10.3747/co.26.4395 31043808 PMC6476465

[14] Guerin PJ Singh-Phulgenda S Strub-Wourgaft N. The consequence of COVID-19 on the global supply of medical products: Why Indian generics matter for the world? F1000Research (2020) 9:225. doi: 10.12688/f1000research.23057.1 32566139 PMC7284150

[15] Aljadeeah S Michielsen J Ravinetto R Hargreaves S Wirtz V Razum O. Facilitating access to medicines and continuity of care for Ukrainian refugees: exceptional response or the promise of more inclusive healthcare for all migrants? BMJ Glob Health (2022) 7(8):e010327. doi: 10.1136/bmjgh-2022-010327 PMC9422850 36008046

